# WY-14643, a Potent Peroxisome Proliferator Activator Receptor-***α*** PPAR-***α*** Agonist Ameliorates the Inflammatory Process Associated to Experimental Periodontitis

**DOI:** 10.1155/2010/193019

**Published:** 2010-12-27

**Authors:** Enrico Briguglio, Rosanna Di Paola, Irene Paterniti, Emanuela Mazzon, Giacomo Oteri, Giancarlo Cordasco, Salvatore Cuzzocrea

**Affiliations:** ^1^Istituto Policattedra di Odontostomatologia, Universita degli Studi di Messina, 98100 Messina, Italy; ^2^IRCCS Centro Neurolesi “Bonino-Pulejo”, 98100 Messina, Italy; ^3^Department of Clinical and Experimental Medicine and Pharmacology, School of Medicine, University of Messina, Torre Biologica, Policlinico Universitario Via C. Valeria, Gazzi, 98100 Messina, Italy

## Abstract

We have investigated the effects of WY14643, a potent peroxisome proliferator activator receptor-*α* (PPAR-*α*) agonist, in a rat model of ligature-induced periodontitis. Male Sprague-Dawley rats were lightly anaesthetized with pentobarbitone (35 mg/kg). Sterile, 2-0 black braided silk thread was placed around the cervix of the lower left first molar and knotted medially. Animals received WY14643 (1 mg/kg i.p, daily for eight days). Eighths days after placement of the ligature, we evaluated several markers of inflammation such us (1) myeloperoxidase activity, (2) a cytokines and adhesion molecules expression, (3) NF-*κ*B expression, (4) iNOS expression, (5) the nitration of tyrosine residues, (6) activation of the nuclear enzyme poly(ADP-ribose) polymerase, (7) apoptosis, and (8) the degree of gingivomucosal tissues injury. Administration of WY14643 significantly decreased all of the parameters of inflammation as described above. These results demonstrate that WY14643 exerts an anti-inflammatory role during experimental periodontitis and is able to ameliorate the tissue damage.

## 1. Introduction

Periodontitis is an inflammatory lesion, mediated by accumulation of bacteria that leads to the loss of connective tissue attachment to root surface cementum and adjacent alveolar bone resulting in tooth loss [1]. Studies have demonstrated that periodontal disease affects between 10% and 15% of the world's population [2]. The exact mechanism of periodontitis development, including the prior agents or mediators involved, is not clear. Periodontitis manifests itself as a multifactor phenomenon. It is now clear that, while the etiology of periodontitis is bacteria, the pathogenesis is inflammatory. 

It is widely accepted that the initiation and progression of periodontitis are dependent upon the presence of microorganisms capable of causing disease. Although more than 500–600 species of microorganisms have been isolated from periodontal pockets [3], it is likely that only a small percentage of these are etiologic agents [4]. In addition to the possible direct pathologic effects of bacteria on the periodontal tissues, it is clear that damage to the periodontium must also occur by indirect means. Bacterial products must gain access to the cellular constituents of the gingival tissues and activate cellular processes that are destructive to collagenous connective tissue and bone [5]. Then, Periodontitis is seen as the direct consequence of bacterial invasion and is regarded as an infectious disease. Recently, however, there arises a question but, microorganisms are the initiators of periodontal disease or are the result? Hasturk et al. in a study of experimental periodontitis, showed that the pharmacological control of the inflammatory process has resulted in the disappearance of the offending organism as an initiator of the disease [6]. 

In this study, we therefore wanted to study whether the modulation of the inflammatory process could limit the development of periodontitis. Peroxisome proliferator-activated receptors (PPARs) are a family of nuclear receptors comprising three isoforms, PPAR*α*, PPAR*δ*, and PPAR*γ*, which act as ligand-activated transcriptional factors. PPARs play key roles in energy homeostasis by modulating glucose and lipid metabolism and transport. PPAR*α* is also critical in inflammation and is the molecular target of the fibrates class of drugs, such as fenofibrate, which act as agonistic ligands of PPAR-*α* [7]. It has been shown that PPAR-*α* ligands regulates inflammatory responses [8]. In particular, it has been reported that PPAR-*α* ligands can inhibit the expression of various proinflammatory genes, such as interleukin (IL)-6, vascular cell adhesion molecule-1, platelet-activating factor (PAF) receptor and cyclooxygenase (COX)-2 (generating PGE_2_ and TxB_2_), in response to cytokine activation [9, 10]. This may, in part, be dependent on the inhibition of functional nuclear factor (NF)-*κ*B activation and on the increase of expression of the inhibitory protein I*κ*B*α* [11]. The present study was carried out in order to gain a better understanding of the possible influence of PPAR-*α* in a mouse model of periodontitis.

## 2. Methods

### 2.1. Surgical Procedure

Male Sprague Dawley rats (280–400 g) were lightly anaesthetized with surgical doses of sodium pentobarbitone (35 mg/kg). Sterile, 2-0 black braided silk thread was placed around the cervix of the lower left first molar and knotted medially as previously described [12]. After the rats had recovered from the anaesthetic they were allowed to eat commercial laboratory food and drink tap water ad libitum. Animal care and protocol was in compliance with Italian regulations on protection of animals used for experimental and other scientific purpose (D.M. 116192) as well as with the EEC regulations (O.J. of E.C. L 358/1 12/18/1986). The animals and the study protocol were approved by the Institutional Animal Care and User Committee of the University of Messina.

### 2.2. Experimental Groups

Rats were randomly allocated into the following groups.

- *Ligature* + *vehicle group*: rats were subjected to ligature-induced periodontitis and animals received vehicle i.p. (1 h after the ligature placement and daily treatment for eight days).
- *Ligature* + *WY14643 group*: rats were subjected to ligature-induced periodontitis and animals received WY14643 (1 mg/kg i.p., 1 h after the ligature placement and daily for eight days).

At 8 days after the ligature induction of periodontitis the rats (*N* = 10 from each group for each parameter) were sacrificed in order to assess the effects of the compound on an acute lesion. The right side that is not subject to ligature, was used as control. 

 The dose of WY14643 was chosen on the basis of previous studies [13].

### 2.3. Histological Examination

For histopathological examination, biopsies of gingiva and mucosa tissue from the buccal and lingual aspect of the teeth were taken 8 days after the ligature induction of periodontitis. The tissue slices were fixed in 10% neutral-buffered formaldehyde for 5 days, embedded in paraffin, and sectioned. The sections, orientated longitudinally from the teeth crowns, were stained with trichrome and haematoxylin eosin stains. In the gingivomucosal sections stained with trichrome stain the total number of infiltrating leukocytes (e.g., neutrophils and mononuclear cells) in cortical interstitial spaces from gingiva and mucosa tissues was assessed quantitatively by counting the number of infiltrating leukocytes in 20 high power fields.

### 2.4. Radiography

Mandibles were placed on a radiographic box at a distance of 90 cm from the X-ray source. Radiographic analysis of normal and ligated mandibles was performed by X-ray machine (Philips X12 Germany) with a 40 kW exposure for 0.01 sec. A radiographic examination of at eight day after ligature placement revealed bone matrix resorption in the lower first left after ligation as previously described [12].

### 2.5. Myeloperoxidase Activity

Myeloperoxidase activity, an indicator of polymorphonuclear leukocyte (PMN) accumulation, was determined as previously described [14]. Gingivomucosal tissue, collected at the specified time, were homogenized in a solution containing 0.5% hexa-decyl-trimethyl-ammonium bromide dissolved in 10 mM potassium phosphate buffer (pH 7) and centrifuged for 30 min at 20,000 × g at 4°C. An aliquot of the supernatant was then allowed to react with a solution of tetramethyl-benzidine (1.6 mM) and 0.1 mM H_2_O_2_. The rate of change in absorbance was measured spectrophotometrically at 650 nm. Myeloperoxidase activity was defined as the quantity of enzyme degrading 1 *μ*mol/min of peroxide at 37°C and was expressed in milliunits/g of wet tissue.

### 2.6. Immunohistochemical Localization of iNOS, TNF-*α*, IL-1*β*, ICAM-1, Nitrotyrosine, PAR, Bax, and Bcl-2

At 8 days after the ligature induction of periodontitis, the gingivomucosal tissues were fixed in 10% buffered formaldehyde and 8 *μ*m sections were prepared from paraffin embedded tissues. After deparaffinization, endogenous peroxidase was quenched with 0.3% H_2_O_2_ in 60% methanol for 30 min. The sections were permeabilized with 0.1% Triton X-100 in PBS for 20 min. Nonspecific adsorption was minimized by incubating the section in 2% normal goat serum in phosphate buffered saline for 20 min. Endogenous biotin or avidin binding sites were blocked by sequential incubation for 15 min with avidin and biotin. The sections were then incubated overnight with primary antiiNOS antibody (Santa Cruz Biotechnology, 1 : 500 in PBS, v/v), antiICAM-1 antibody (BD Pharmingen, CD54, 1 : 500), antinitrotyrosine rabbit polyclonal antibody (1 : 500 in PBS, v/v), antipoly(ADP-ribose) goat, an indicator of PARP activation, (PAR; 1 : 500 in PBS, v/v) (Alexis; DBA, Milan, Italy) antiBax polyclonal antibody (Santa Cruz Biotechnology, 1 : 500 in PBS, v/v),with antiBcl-2 polyclonal antibody (Santa Cruz Biotechnology), anti TNF-*α* polyclonal antibody (Santa Cruz Biotechnology, 1 : 500 in PBS, v/v), anti IL-1*β* polyclonal antibody (Santa Cruz Biotechnology, 1 : 500 in PBS, v/v) or with control solutions. Controls included buffer alone or nonspecific purified rabbit IgG. Specific labeling was detected with a biotin-conjugated goat antirabbit IgG and avidin-biotin peroxidase complex (DBA, Milan, Italy). The counter stain was developed with DAB (brown color) and nuclear fast red (red background). A positive staining (brown color) was found in the sections, indicating that the immunoreactions were positive, no positive staining (pink color) was observed in the sections indicating that the immunoreactions were negative. Immunocytochemistry photographs (N = 5) were assessed by densitometry as by using Optilab Graftek software on a Macintosh personal computer.

### 2.7. Western Blot Analysis for IkB-*α*, iNOS and Caspase-3

In brief, gingivomucosal tissues from each rat were suspended in extraction buffer A containing 0.2 mM phenylmethylsulfonyl fluoride (PMSF), 0.15 *μ*M pepstatin A, 20 *μ*M leupeptin, and 1 *μ*M sodium orthovanadate; homogenized at the highest setting for 2 min; and centrifuged at 1000 g for 10 min at 4°C. Supernatants represented the cytosolic fraction. The pellets, containing enriched nuclei, were resuspended in buffer B containing 1% Triton X-100, 150 mM NaCl, 10 mM Tris-HCl, pH 7.4, 1 mM EGTA, 1 mM EDTA, 0.2 mM PMSF, 20 M leupeptin, and 0.2 mM sodium orthovanadate. After centrifugation at 15,000 g for 30 min at 4°C, the supernatants containing the nuclear protein were stored at −80°C for further analysis. Protein concentration was determined with the Bradford-based kits (Bio-Rad, Milan, Italy). The levels of IkB-*α*, iNOS and caspase-3 were quantified in cytosolic fraction. The filters were blocked with 1 xPBS, 5% (w/v) nonfat dried milk for 40 min at room temperature, and they were subsequently probed with specific antibodies IkB-*α* (1 : 1000; Santa Cruz Biotechnology, Inc.), antiiNOS (1 : 500; Santa Cruz Biotechnology, Inc.), anticaspase-3 (Cell Signaling, 1 : 500), in 1 xPBS, 5% (w/v) nonfat dried milk, and 0.1% Tween 20 at 4°C overnight. Membranes were incubated with peroxidase-conjugated bovine antimouse IgG secondary antibody or peroxidase-conjugated goat antirabbit IgG (1 : 2000; Jackson Immuno Research Laboratories Inc., West Grove, PA, USA) for 1 h at room temperature. The relative expression of the protein bands of IkB-*α* (~37 kDa), iNOS (~135 kDa) and caspase-3 (cleaved caspase 3 (~17 kDa) was quantified by densitometric scanning of the X-ray films with GS-700 imaging densitometer (GS-700; Bio-Rad, Milan, Italy) and a computer program (Molecular Analyst, IBM).

### 2.8. Materials

The primary antibodies directed at Bax and Bcl-2 was obtained from Santa Cruz Biotechnology, Inc. (Santa Cruz, CA, USA). The secondary antibody was obtained from Jackson Immuno Research, Laboratories, Inc. (Jackson, Bar Harbor, Maine, USA). Unless otherwise stated, all compounds were obtained from Sigma-Aldrich Company Ltd (Milan, Italy). All other chemicals were of the highest commercial grade available. All stock solutions were prepared in nonpyrogenic saline (0.9% NaCl; Baxter, Italy, UK).

### 2.9. Data Analysis

All values in the Figures and text are expressed as mean ± standard error of the mean of *n* observations, where *n *represents the number of animals studied. Data sets were examined by one-and two-way analysis of variance and individual group means were then compared with Bonferroni or Student's unpaired *t*-test. A *P*-value less than .05, was considered significant. In the experiments involving histology or immunohistochemistry, the Figures shown are representative of at least 3 experiments performed on different experimental days.

## 3. Results

### 3.1. Effect of WY14643 on Tissue Damage and Bone Resorption

**Figure 1:**
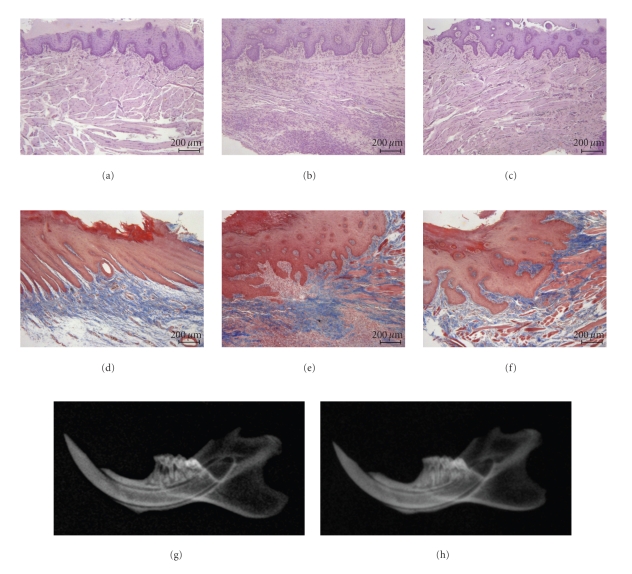
Effect of WY14643 on tissue damage and bone resorption. Gingivomucosal section from contra lateral side obtained from vehicle-treated rats (a) demonstrating no tissue damage. Inflammatory cells infiltration and edema was observed in gingivomucosal section from ligature-treated rats. (b) Significantly less edema and inflammatory cells infiltration was observed in gingivomucosal section from ligature-treated rats which have been treated with WY14643. (c) Moreover Masson's trichrome stain was negative in gingivomucosal tissue sections taken from the contra lateral side (d) from vehicle when compared with gingivomucosal tissues sections of ligature-operated rats. (e) WY14643 treatment reduced the increase of collagen. (f) The alveolar bone from ligated rats demonstrated alveolar bone resorption. (g) WY14643 treatment suppressed alveolar pathology in the rat alveolar bone. (h) Figure is representative of at least 3 experiments performed on different experimental days.

When compared to gingivomucosal tissues sections taken from the contra lateral side obtained from vehicle-treated rats (Figure 1(a)), histological examination of gingivomucosal tissues sections of ligature-operated rats showed oedema, tissue injury as well as infiltration of the tissue with inflammatory cells (Figure 1(b)). WY14643 treatment reduced the degree of gingivomucosal tissues injury (Figure 1(c)). Moreover Masson's trichrome stain, which is used to monitor the increase of collagen fiber, was negative in gingivomucosal tissue sections taken from the contra lateral side from vehicle when compared with gingivomucosal tissues sections of ligature-operated rats (Figures 1(d) and 1(e), resp.). WY14643 treatment reduced the increase of collagen (Figure 1(f)). In addition a radiographic examination of the mandibles, at eight day after ligature placement, revealed bone matrix resorption in the lower left first molar region after ligation (Figure 1(g)). There was no evidence of pathology in right first molar (data not shown). WY14643 markedly reduced the degree of bone resorption in the lower left first molar region after ligation (Figure 1(h)).

### 3.2. Effects of WY14643 Treatment on NF-*κ*B Activation in Periodontitis

**Figure 2:**
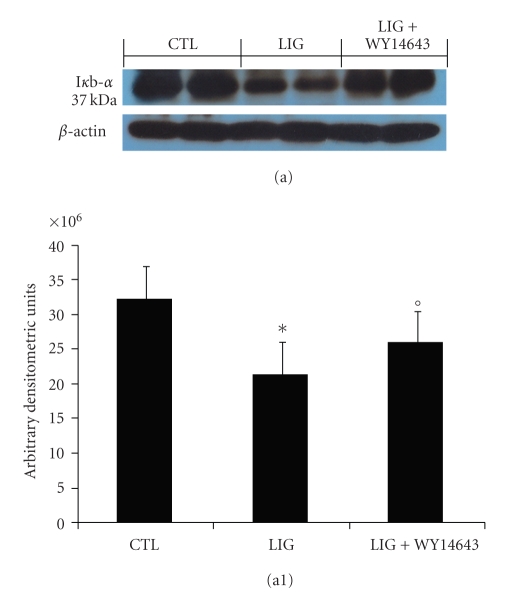
Effects of WY14643 treatment on NF-*κ*B activation in periodontitis. Representative Western blots showing the effects of WY14643 on I*κ*B-*α* degradation (a, a1) in the gingivomucosal tissues from ligature-operated rats. A representative blot of lysates (a) obtained from 5 animals per group is shown and densitometry analysis of all animals is reported. The results in panel (a1) are expressed as mean ± s.e.m. from *n* = 5/6 gingivomucosal tissues for each group. *P < .01 versus nonligated. °*P* < .01 versus ligated.

We evaluated I*κ*B-*α* degradation by Western Blot analysis to investigate the cellular mechanisms by which treatment with WY14643 may attenuate the development of periodontitis. A basal level of I*κ*B-*α* was detected in the gingivomucosal tissues from the contralateral side obtained from vehicle-treated rats, whereas in the gingivomucosal tissues from ligature-operated rats I*κ*B-*α* levels were substantially reduced (Figures 2a and 2a1). WY14643 treatment prevented the ligature induced I*κ*B-*α* degradations in the gingivomucosal tissues at eight days following ligation (Figures 2a and 2a1).

### 3.3. Effect of WY14643 on Cytokines Expression

**Figure 3:**
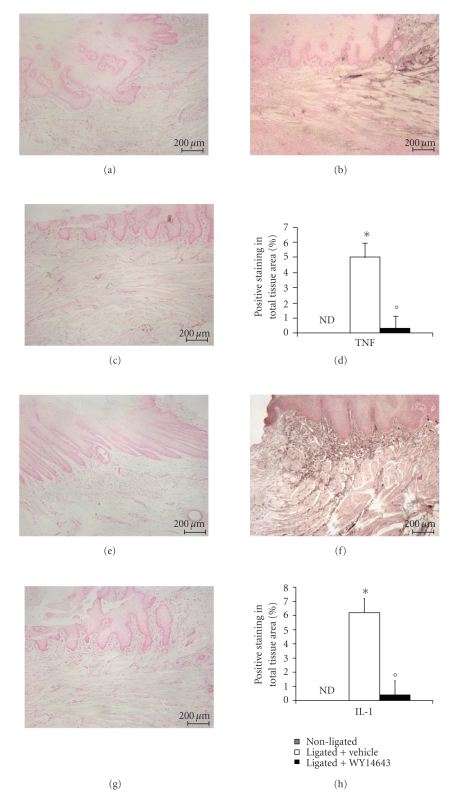
Effect of WY14643 on cytokines expression. Immunohistochemical analysis of gingivomucosal tissues from the contra lateral side obtained from vehicle-treated rats did not reveal any immunoreactivity for TNF-*α* and IL-1*β* (a, e). Positive staining for for TNF-*α* and IL-1*β* were found in the gingivomucosal tissues from ligature operated rats (b, f) after ligature. WY14643 treatment significantly reduced the degree of positive staining for TNF-*α* and IL-1*β* (c, g). Densitometry analysis of immunocytochemistry photographs (d, h); *n* = 5 photos from each samples collected from all rats in each experimental group) for TNF-*α* and IL-1*β* from gingivomucosal tissue was assessed. The assay was carried out by using Optilab Graftek software on a Macintosh personal computer (CPU G3-266). Densitometry data are expressed as % of total tissue area.

To test whether WY14643 modulates the inflammatory process through the regulation of secretion of proinflammatory cytokines, we analyzed, by immunohistochemical analysis, levels of TNF-*α* and IL-1*β*. Immunohistochemical analysis of gingivomucosal tissues from the contra lateral side obtained from vehicle-treated rats did not reveal any immunoreactivity for TNF-*α* and IL-1*β* (Figures 3(a) and 3(e), resp., see densitometry D, H). In contrast, 8 days following ligation, positive staining for TNF-*α* and IL-1*β* were found in the gingivomucosal tissues from ligature operated rats (Figures 3(b) and 3(f), resp., see densitometry D, H). WY14643 treatment significantly reduced the degree of positive staining for these proinflammatory cytokines TNF-*α* and IL-1*β* (Figures 3(c) and 3(g), resp., see densitometry D, H).

### 3.4. Effects of WY14643 on the Expression of Adhesion Molecules (ICAM-1) and Neutrophils Infiltration in Periodontitis

**Figure 4:**
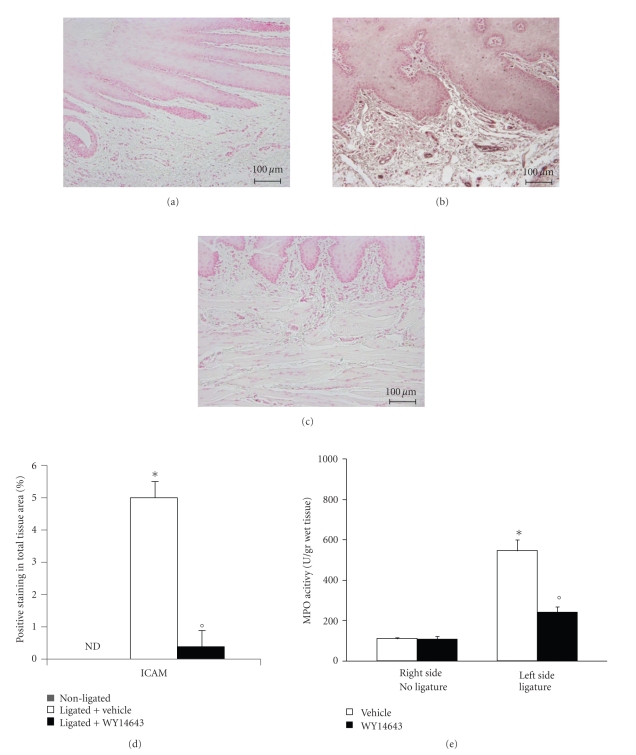
Effects of WY14643 on the expression of ICAM-1 and neutrophils infiltration. Immunohistochemical analysis of gingivomucosal tissues from the contra lateral side obtained from vehicle-treated rats did not reveal any immunoreactivity for ICAM-1. (a) Positive staining for ICAM-1 was found in the gingivomucosal tissues from ligature operated rats. (b) In contrast, a positive staining for ICAM-1 was significantly attenuated by the treatment with WY14643. (c) Moreover myeloperoxidase activity (e) in gingivomucosal tissue was significantly increased by ligature compared to the contra lateral side obtained from vehicle-treated rats. WY14643 significantly reduced myeloperoxidase activity levels. Figure is representative of at least 3 experiments performed on different experimental days. Densitometry analysis of immunocytochemistry photographs; (d) *n* = 5 photos from each samples collected from all rats in each experimental group) for ICAM-1 from gingivomucosal tissue was assessed. The assay was carried out by using Optilab Graftek software on a Macintosh personal computer (CPU G3-266). Densitometry data are expressed as % of total tissue area. Data are means of mean ± s.e.m. from *n* = 10 rats for each group. *P < .01 versus nonligated. °*P* < .01 versus ligated.

Immunohistochemical analysis of gingivomucosal tissues from the contra lateral side obtained from vehicle-treated rats did not reveal any immunoreactivity for ICAM-1 (Figure 4(a) see densitometry D). In contrast, 8 days following ligation, positive staining for ICAM-1, was found in the gingivomucosal tissues from ligature operated rats (Figure 4(b) see densitometry D), mainly localized in the inflammatory cells in derma and around the vessels. In contrast, a positive staining for ICAM-1 was significantly attenuated by the treatment with WY14643 (Figure 4(c) see densitometry D). In addition myeloperoxidase activity was significantly elevated at eight days after the ligature (Figure 4(e)) and WY14643-treatment significantly reduced these levels (Figure 4(e)). No significant changes of myeloperoxidase activity were observed in the gingivomucosal tissues from the contra lateral side obtained from vehicle-treated rats (Figure 4(e)).

### 3.5. Effects of WY14643 on iNOS Expression in Periodontitis

**Figure 5:**
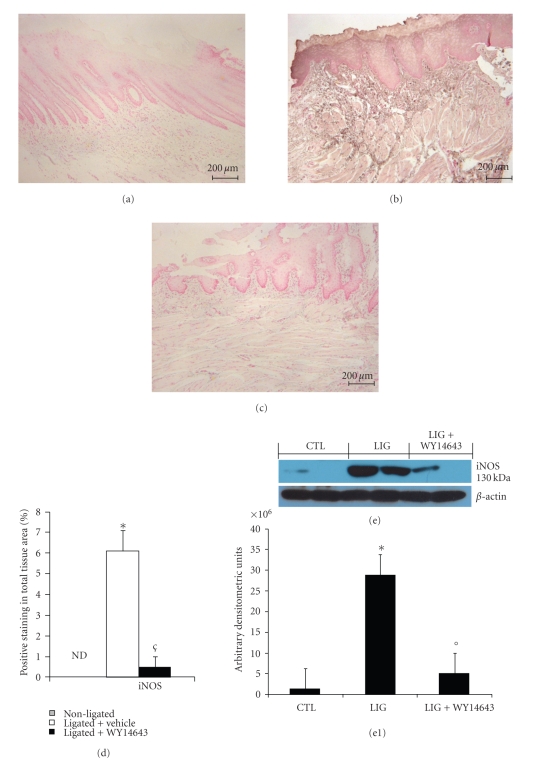
Effect of WY14643 on iNOS expression. Sections of gingivomucosal tissues from the contra lateral side obtained from vehicle-treated rats did not reveal any immunoreactivity for iNOS. (a) Positive staining for iNOS (b) was observed in gingivomucosal tissue after ligature. In gingivomucosal tissue of WY14643 treated rats no positive staining was observed for iNOS. (c) Densitometry analysis of immunocytochemistry photographs; ((d) *n* = 5 photos from each samples collected from all rats in each experimental group) for iNOS from gingivomucosal tissue was assessed. The assay was carried out by using Optilab Graftek software on a Macintosh personal computer (CPU G3-266). Densitometry data are expressed as % of total tissue area. Moreover, a significant increase in iNOS expression (e, e1), assayed by Western blot analysis, was detected in in gingivomucosal tissue after ligature. Treatment with WY14643 significantly attenuated iNOS (e, e1) expression in gingivomucosal tissue after ligature. A representative blot of lysates (e, e1) obtained from 5 animals per group is shown and densitometry analysis of all animals is reported. Figure is representative of at least 3 experiments performed on different experimental days. *P < .01 versus nonligated. °*P* < .01 versus ligated.

Sections of gingivomucosal tissues from the contra lateral side obtained from vehicle-treated rats did not reveal any immunoreactivity for iNOS (Figure 5(a) see densitometry D) within the normal architecture. At eight days following ligation, a positive staining for iNOS (Figure 5(b) see densitometry D) was found in the gingivomucosal tissues from ligature-operated rats. WY14643 abolished the staining for iNOS (Figure 5(c) see densitometry D). Furthermore, ligature caused a significant increase of iNOS expression, assayed by Western blot analysis, in the gingivomucosal tissues from ligature-operated rats compared to the gingivomucosal tissues from the contra lateral side obtained from vehicle-treated rats (Figures 5e and 5e1). A significant reduction of the iNOS levels was observed in the tissues from WY14643 treated rats (Figures 5e and 5e1).

### 3.6. Effects of WY14643 on Nitrotyrosine Formation, and Poly (ADP-Ribose) Polymerase Activation in Periodontitis

**Figure 6:**
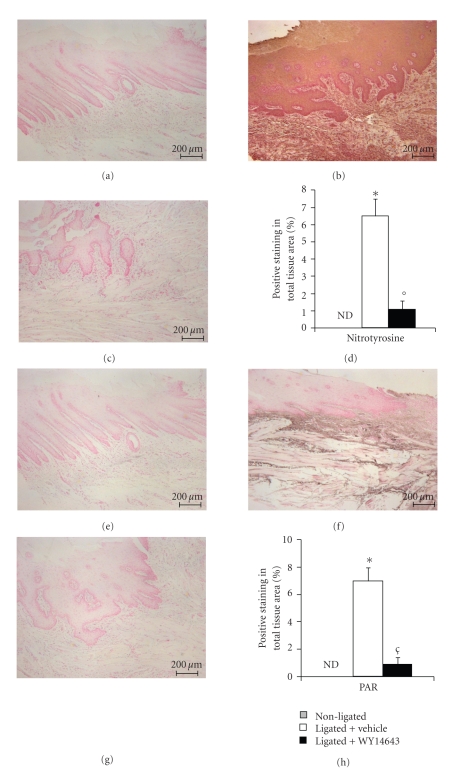
Effect of WY14643 on ligature-induced nitrotyrosine formation and PARP activation. No positive staining for nitrotyrosine was observed in gingivomucosal tissues from the contra lateral side obtained from vehicle-treated rats. (a) Positive staining for nitrotyrosine (b) was observed in gingivomucosal tissue after ligature. In gingivomucosal tissue of WY14643 treated rats no positive nitrotyrosine staining was observed. (c) In addition, immunohistochemistry for PAR, an indicator of *in vivo* PARP activation, revealed the occurrence of positive staining for PAR localized in gingivomucosal tissue after ligature. (f) WY14643 treatment reduced the degree of positive staining for PAR (g) in the gingivomucosal tissue. No positive staining for PAR was observed in gingivomucosal tissues from the contra lateral side obtained from vehicle-treated rats (e). Densitometry analysis of immunocytochemistry photographs ((d, h); *n* = 5 photos from each samples collected from all rats in each experimental group) for nitrotyrosine and PAR from gingivomucosal tissue was assessed. The assay was carried out by using Optilab Graftek software on a Macintosh personal computer (CPU G3-266). Densitometry data are expressed as % of total tissue area. Figure is representative of at least 3 experiments performed on different experimental days. *P < .01 versus nonligated. °*P* < .01 versus ligated.

Nitrotyrosine, a specific marker of NO-dependent oxidative stress, was measured by immunohistochemical analysis in the gingivomucosal tissues sections to determine the localization of *“peroxynitrite formation”* and/or other reactive nitrogen derivatives produced during experimental periodontitis. Sections of gingivomucosal tissues from the contra lateral side obtained from vehicle-treated rats did not reveal any immunoreactivity for nitrotyrosine (Figure 6(a) see densitometry H) within the normal architecture. At eight days following ligation, a positive staining for nitrotyrosine was found in the gingivomucosal tissues from ligature-operated rats (Figure 6(b) see densitometry H). WY14643 treatment abolished the staining for nitrotyrosine (Figure 6(c) see densitometry H). Moreover, in our study, we have evaluated formation of polymer of ADP-ribose (PAR), which is synthesized by PAR polymerases (PARPs) from NAD (+). It as an indicator of *in vivo* PARP activation and regulates cell-survival and cell-death programmes. Immunohistochemistry for PAR, revealed the occurrence of positive staining for PAR in the gingivomucosal tissues from ligature-operated rats (Figure 6(f) see densitometry H). WY14643 treatment reduced the degree of positive staining for PAR (Figure 6(g) see densitometry H) in the in the gingivomucosal tissues. Please note that no positive staining for PAR have been found in gingivomucosal tissues section from the contra lateral side obtained from vehicle-treated rats (Figure 6(e) see densitometry H).

### 3.7. Effects of WY14643 on Bax and Bcl-2 Expression

To test whether PPAR-*α* gene plays a role on apoptosis in gingivomucosal tissues after ligature placement, we measured Bax and Bcl-2 expression by Immunohistochemical analysis at eight days following ligation. 

**Figure 7:**
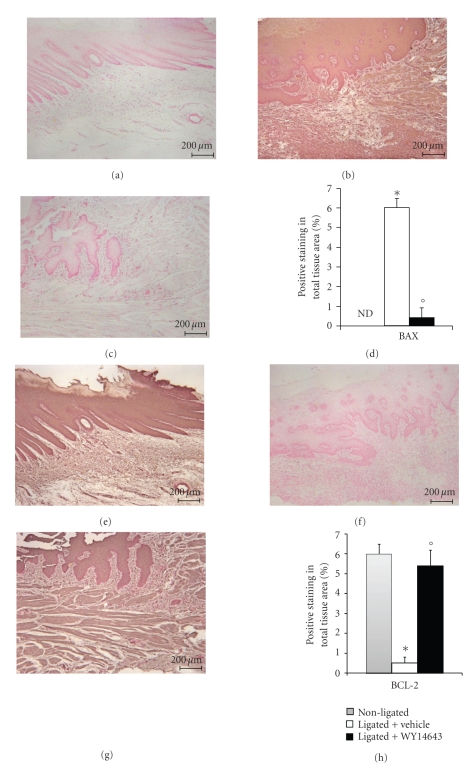
Effects of WY14643 on Bax and Bcl-2 expression. No positive staining for Bax was observed in gingivomucosal tissues from the contra lateral side obtained from vehicle-treated rats. (a) Immunohistochemistry for Bax showed positive staining in the gingivomucosal sections after ligature. (b) The degree of positive staining for Bax was markedly reduced in WY14643 treated rats. (c) In immunohistochemical assay the degree of positive staining for Bcl-2 was markedly reduced in gingivomucosal sections obtained after ligature. (f) The reduction of Bcl-2 expression caused by ligature was significantly attenuated in gingivomucosal sections from WY14643treated rats. (g) A normal basal staining for Bcl-2 was observed in gingivomucosal tissues from the contra lateral side obtained from vehicle-treated rats. (e) Densitometry analysis of immunocytochemistry photographs ((d, h); *n* = 5 photos from each samples collected from all rats in each experimental group) for Bax and Bcl-2 from gingivomucosal tissue was assessed. The assay was carried out by using Optilab Graftek software on a Macintosh personal computer (CPU G3-266). Densitometry data are expressed as % of total tissue area. Figure is representative of at least 3 experiments performed on different experimental days. *P < .01 versus nonligated. °*P* < .01 versus ligated.

**Figure 8:**
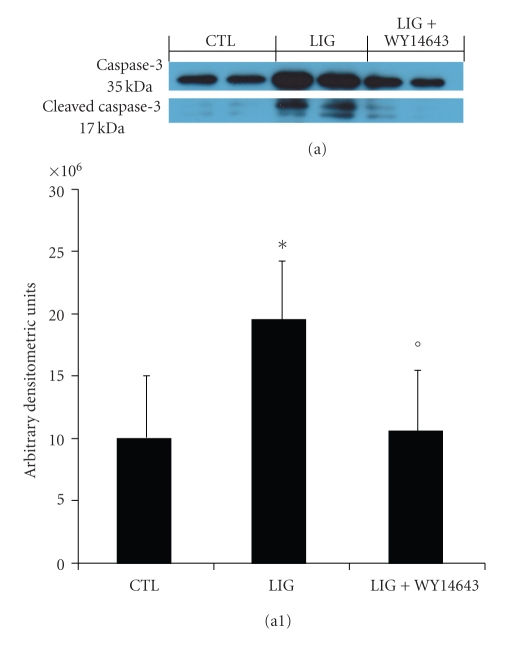
Effects of WY14643 on Cleaved caspase 3 levels. We have evaluated, by western blot analysis, a key mediator of apoptosis belong to the asparate-specific cysteinyl proteases or caspases. No cleaved caspase 3 expression were detectable in the homogenized gingivomucosal tissues from the contra lateral side obtained from vehicle-treated rats (a, a1).Cleaved caspase 3 levels were appreciably increased following ligation (a, a1). Treatment of rats with WY14643 (1 mg/kg) significantly attenuated ligation-induced of Cleaved caspase 3 levels (a, a1). A representative blot of lysates (a, a1) obtained from 5 animals per group is shown and densitometry analysis of all animals is reported. Figure is representative of at least 3 experiments performed on different experimental days. *P < .01 versus nonligated. °*P* < .01 versus ligated.

No positive staining for Bax was observed in gingivomucosal tissues from the contra lateral side obtained from vehicle-treated rats (Figure 7(a)). Immunohistochemistry for Bax showed positive staining in the gingivomucosal sections after ligature (Figure 7(b)). The degree of positive staining for Bax was markedly reduced in WY14643 (1 mg/kg) treated rats (Figure 7(c)). To detect Bcl-2 expression, whole sample from gingivomucosal tissues of rats were also analyzed by immunohistochemical analysis In immunohistochemical assay the degree of positive staining for Bcl-2 was markedly reduced in gingivomucosal sections obtained after ligature (Figure 7(f)). The reduction of Bcl-2 expression caused by ligature was significantly attenuated in gingivomucosal sections from WY14643treated rats (Figure 7(g)). A normal basal staining for Bcl-2 was observed in gingivomucosal tissues from the contra lateral side obtained from vehicle-treated rats (Figure 7(e)). Moreover we have evaluated, by western blot analysis, a key mediator of apoptosis belong to the asparate-specific cysteinyl proteases or caspases. No cleaved caspase-3 expression was detectable in the homogenized gingivomucosal tissues from the contra lateral side obtained from vehicle-treated rats (Figures 8a and 8a1). Cleaved caspase-3 levels were appreciably increased following ligation (Figures 8a and 8a1). Treatment of rats with WY14643 (1 mg/kg) significantly attenuated ligation-induced of Cleaved caspase 3 levels (Figures 8a, a1).

## 4. Discussion

In this study, we demonstrate that a PPAR*α* agonist, WY14643, exerts beneficial effects in a rat model of periodontitis. The treatment with WY14643 attenuates: (1) Myeloperoxidase activity, (2) cytokines and adhesion molecules expression, (3) NF-*κ*B expression, (4) iNOS expression, (5) the nitration of tyrosine residues (6) activation of the nuclear enzyme poly (ADP-ribose) polymerase, (7) apoptosis (8) the degree of gingivomucosal tissues in rats subjected to ligature-induced periodontitis. All of these findings support the view that PPAR*α* has a detrimental role in the development of injury associated with periodontitis in rats. It has been known that PPAR*α* agonists have antiinflammatory characteristics [15]. Although much is known about gene activation by PPARs acting via PPREs, less information exists about the mechanisms of negative gene modulation by PPARs. Recently, PPAR*α* have been suggested to exert antiinflammatory activities interfering negatively with other transcription factor pathways such as NF-*κ*B, signal transducers and activators of transcription (STAT), and activator protein-1 (AP-1) [15]. PPAR-*α* increases I*κ*B*α* expression, thus preventing nuclear p50/p65 NF-*κ*B translocation and arresting their nuclear transcriptional activity. These findings identify a molecular mechanism of negative gene regulation by PPAR-*α* and reveal a direct implication for PPAR-*α* in the modulation of the inflammatory gene response in inflammation. In this work we demonstrate that the WY 14643 treatment inhibited I*κ*B-*α* degradation in periodontal tissue. NF-*κ*B activation induces the transcription of many proinflammatory genes, including nitric oxide synthase expression (iNOS), TNF-*α*, IL-1*β*, and ICAM-1, to name but a few [16]. In precedent study we have demonstrated that, in periodontitis, iNOS expression have a detrimental effects such us a cytotoxic action toward the host tissues, alveolar bone resorption due to the stimulating effect of nitric oxide on the activity of the osteoclasts [12, 17]. In this study we determined the expression, and thus the formation, of iNOS, through the technique of immunohistochemistry. the same technique was used to determine the localization of iNOS in biopsies from patients with periodontitis [18]. Our results demonstrate that WY 14643 treatment attenuates the expression of iNOS in periodontal tissue. During the initiation and progression of periodontal disease, inflammatory cytokines are considered to play important roles. Several reports have suggested a relationship between the progression of periodontitis and the expression of interleukin-1 (IL-1), IL-6, IL-8 and tumour necrosis factor-*α* in gingival tissues [19]. We confirm that the model of periodontitis used here leads to a substantial increase in the levels of TNF-*α* and IL-1*β* in the gingivomucosal tissue. Interestingly, the levels of these two proinflammatory cytokines are significantly lower in the ligated-rats that were treated with WY 14643. In addition, we also report in the present study that ligature-induced periodontitis in the rat results in a significant infiltration of inflammatory cells in the gingivomucosal tissues and we also demonstrated that treatment with WY 14643 reduces this inflammatory cells infiltration as assessed by myeloperoxidase and with the moderation of the tissue damage as evaluated by histological examination. A possible mechanism, by which WY 14643 attenuates polymorphonuclear cells infiltration, is by downregulating adhesion molecules ICAM-1 as demonstrated here [20]. These findings, therefore, suggest that exogenous PPAR-*α* ligand reduced the activation and the subsequent expression of proinflammatory genes. Activation and recruitment of neutrophils and macrophages, in periodontitis, results in the subsequently release mediators including reactive oxygen species (ROS) [21] and nitric oxide (NO) [22] involved in a variety of inflammatory conditions [23, 24]. 

There are some evidences that NO inhibits neutrophil migration by a mechanism dependent on the expression of ICAM-1 on mesenteric microcirculation vessels of mice subjected to experimental acute peritonitis by an injection of either LPS, carrageenan (Cg), or (fMLP) [25]. In this study, the authors treated the experimental peritonitis in mice with chemical inhibitors for NO synthase (NOS) and demonstrated an increase in the migration of neutrophils into venular endothelium and enhanced the expression of ICAM-1 on the endothelium. Interesting PPAR*γ* agonists suppressed neutrophils migration in a NO expression dependent manner via iNOS which in turn suppress the ICAM-1 expression by endothelial cells [26]. Also, agonists for PPAR-*γ* appear to amplify iNOS expression while they inhibit the activation of the inflammatory signal transduction molecule, NF-*κ*B [27]. 

In our previous study we have just demonstrated that a PPAR-*γ* agonist, as rosiglitazone, reduced the cellular infiltration into the gingivomucosal tissue, in a experimental model of periodontitis induced by ligature. A possible mechanism by which rosiglitazone attenuates polymorphonuclear cell infiltration is by downregulating adhesion molecules ICAM-1 and P-selectin expression in a NO dependent manner [28]. Several studies also support the conclusion that NO from iNOS plays an important role in the pathogenesis of periodontitis [12]. Our finding of reduced iNOS expression by rosiglitazone in vitro is also in accordance with our recent reports clearly demonstrating that rosiglitazone inhibits the expression of iNOS in another model of inflammation. 

Thus, we also demonstrated in previous study such as in experimental model a inflammatory bowel disease [29], that PPAR-*α* agonist was able to reduce neutrophil infiltration in a NO expression dependent manner via iNOS. 

Several studies have implicated the role of ROS and reactive nitrogen species in tissue destruction associated with inflammatory periodontal diseases [30] via several mechanisms including peroxidation of membrane lipids, protein denaturation and DNA damage. To confirm the pathological contributions of peroxynitrite to tissue damage after ligature, we evaluated nitrotyrosine formation, an index of “increased nitrosative stress” in the injured tissue. We observed that the immunostaining for nitrotyrosine is reduced in rats treated with WY 14643. ROS produce strand breaks in DNA that triggers energy-consuming DNA repair mechanisms and activates the nuclear enzyme PARP resulting in the activation of “*the PARP Suicide Hypothesis*”. There is recent evidence that the activation of PARP may also play an important role in experimental periodontitis [12, 31]. We demonstrate here that WY 14643 attenuates the increase in PARP activity in the periodontal tissue. Apoptosis, or programmed cell death, is a form of physiological cell death. It is increased or decreased in the presence of infection, inflammation or tissue remodelling. Previous studies have suggested that apoptosis is involved in the pathogenesis of inflammatory periodontal disease [32]. It has also been demonstrated that the higher frequency of Bcl-2 expression results in progressive periodontal destruction [33]. As apoptosis is an exceedingly complex process involving a large variety of signalling molecules, we have focused our attention on a few selective major players. From the results, we identified proapoptotic transcriptional changes, including up regulation of proapoptotic Bax and downregulation of antiapoptotic Bcl-2, using a Western blot assay. This is the first study to show that treatment with WY 14643 in periodontitis and prevents the loss of the antiapoptotic pathway and, also, reduces activation of the proapoptotic pathway by an, as yet, unidentified mechanism.

## 5. Conclusion

In conclusion, this study provides the first evidence that WY 14643 causes a substantial reduction of ligature-induced periodontitis in the rat. The mechanisms underlying the protective properties of WY14643 involve modulation of transcription factors and consequent altered gene expression, resulting in downregulation of inflammation. These findings provide support that WY14643 may provide a promising approach for the treatment of oxidative stress-related neurodegenerative diseases.

##  Conflict of Intersts 

None of the authors have financial or other conflicts of interests to disclose.
